# The influence of cathelicidin LL37 in human anti-neutrophils cytoplasmic antibody (ANCA)-associated vasculitis

**DOI:** 10.1186/ar4344

**Published:** 2013-10-24

**Authors:** Ying Zhang, Weiwei Shi, Sha Tang, Jingyi Li, Shiwei Yin, Xuejing Gao, Li Wang, Liyun Zou, Jinghong Zhao, Yunjian Huang, Lianyu Shan, Abdelilah S Gounni, Yuzhang Wu, Fahuan Yuan, Jingbo Zhang

**Affiliations:** 1 Department of Nephrology, Xinqiao Hospital, Third Military Medical University, Chongqing 400037, China; 2 Institute of Immunology of PLA, Third Military Medical University, Chongqing 400038, China; 3 Department of Immunology, University of Manitoba, Faculty of Medicine, Winnipeg, Manitoba R3E 0 T5, Canada

## Abstract

**Introduction:**

Antineutrophil cytoplasmic antibody (ANCA)-associated vasculitis (AAV) is characterised by the autoinflammation and necrosis of blood vessel walls. The renal involvement is commonly characterised by a pauci-immune crescentic glomerulonephritis (PiCGN) with a very rapid decline in renal function. Cathelicidin LL37, an endogenous antimicrobial peptide, has recently been implicated in the pathogenesis of autoimmune diseases. To assess whether serum LL37 reflects renal crescentic formation, we measured the serum levels of LL37 in AAV patients with and without crescentic glomerulonephritis (crescentic GN) as compared to healthy controls (HCs). We also analysed the correlation of the serum levels of LL37 and interferon-α (IFN-α) with the clinical characteristics of the patients.

**Methods:**

The study population consisted of 85 AAV patients and 51 HCs. In 40 ANCA-positive patients, a parallel analysis was performed, including the assessment of LL37 and IFN-α levels in the serum and renal biopsies. Of those studied, 15 AAV patients had biopsy-proven crescentic GN, and 25 AAV patients lacked crescent formation. The serum levels of cathelicidin LL37 and IFN-α were both measured by ELISA, and the clinical and serological parameters were assessed according to routine procedures. Immunofluorescence staining was performed on frozen sections of kidney needle biopsies from AAV patients with crescentic GN.

**Results:**

The serum levels of LL37 and IFN-α were significantly increased in AAV patients with crescentic GN compared to AAV patients without crescentic formation and HCs, and patients with high LL37 and IFN-α levels were more likely to be in the crescentic GN group. The LL37 levels were positively correlated with the IFN-α levels, and both LL37 and IFN-α levels showed a positive correlation with serum creatinine and no correlation with complement C3. The renal tissue of crescentic GN patients showed expression of LL37 and IFN-α at the Bowman’s capsule and extracellular sites, suggesting the active release of LL37 and IFN-α.

**Conclusions:**

Significantly higher levels of LL-37 and IFN-α were observed in AAV patients, particularly those with crescentic formation, and LL37 and IFN-α were expressed in the renal tissue of patients with crescentic GN. These data suggest that serum levels of LL37 and IFN-α may reflect both local renal inflammation and systemic inflammation.

## Introduction

Anti-neutrophil cytoplasm antibody (ANCA)-associated vasculitis (AAV) represents a group of systemic autoimmune diseases including Wegener’s granulomatosis, microscopic polyangiitis, Churg–Strauss syndrome and renal limited vasculitis [1]. Renal involvement is frequently manifested as focal segmental necrotising glomerulonephritis (GN), typically pauci-immune crescentic glomerulonephritis (PiCGN). Myeloperoxidase (MPO) and proteinase-3 (PR3) have been identified as targets of classical ANCA and have proved invaluable for the diagnosis and monitoring of disease activity [1]. In addition, the presence of autoantibodies to lysosomal membrane protein-2 (LAMP-2) represents an additional ANCA subtype [2-4]. However, the mechanisms underlying the pathogenesis of PiCGN remain elusive. As an endogenous antimicrobial peptide, LL37 has recently been implicated in the pathogenesis of autoimmune diseases [5]. Autoinflammatory conditions such as psoriasis and systemic lupus erythematosus can be driven by plasmacytoid dendritic cells (pDCs), which produce large amounts of interferon alpha (IFNα) in the presence of DNA and cathelicidin LL37 [6,7]. Recently, DNA-containing LL37 was shown to be involved in the renal damage in AAV, with increased concentrations of IFNα in serum samples from individuals with active AAV [8].

Cathelicidin LL37 is a member of an antimicrobial peptide family found within the lysosomes of macrophages, and polymorphonuclear leukocytes serve a critical role in immune defence against invasive bacteria [9]. For example, neutrophil extracellular traps (NETs) are a unique method by which neutrophils can cause cell death via the release of a meshwork of chromatin fibres decorated with granule-derived antimicrobial peptides; however, these NETs are a potential source of auto-antigens and may contribute to organ damage and vascular disease [10]. These NET-derived constituents stimulate pDCs to release IFNα, which establishes a positive feedback loop in which NETs stimulate IFNα release from pDCs, and this cytokine then primes neutrophils for additional NET formation [6,10,11].

In this study, we hypothesised that the serum levels of LL37 would reflect systemic and renal local inflammation in patients with PiCGN. Therefore, we investigated LL37 and IFNα levels using enzyme-linked immunosorbent assays (ELISAs) in AAV patients and correlated these results to the serological parameters assessed.

## Methods

### Patients

Adult subjects with AAV, who were diagnosed according to the Chapel Hill definition [12], were recruited from the Department of Nephrology, Xinqiao Hospital, Third Military Medical University. The patient characteristics are presented in Table 1. Serum samples were collected from the patients and healthy controls (HCs). Among the 85 patients, 40 patients underwent renal biopsies concomitantly obtained with the serum samples, and all biopsies were reviewed and classified by an experienced nephropathologist according to the revised criteria for PiCGN. Criteria used in the study to define PiCGN by immunofluorescence analysis for frozen tissue sections presented deficiency of immunoglobulin and complements. PR3-ANCA and MPO-ANCA were evaluated using EUROBlot kits (DL-1200-6421-3G; Euroimmun;Lübeck, Germany) and indirect immunofluorescence (FA-1200-2010; Euroimmun; Lübeck, Germany) according to the protocol provided by the manufacturer. The levels of creatinine, blood urea nitrogen and complement factor C3 in the sera were determined using routine techniques. Crescentic GN was defined as crescents in >50% glomeruli. Frozen sections of renal biopsy specimens obtained from 40 patients, among of them, 15 patients with crescentic GN and 25 patients without crescentic GN were included in the present study. The study protocols were approved by the ethics committee board of Xinqiao Hospital, and all subjects gave written informed consent.

**Table 1:** Clinical characteristics of patients with sera included in the cohort

	**Crescentic-positive AAV**	**Crescentic-negative AAV**	**Without renal biopsy**
Total	15	25	45
Male/female	7/8	9/16	19/26
Age (years)	61 (8 to 78)	44 (7 to 76)	55 (20 to 78)
MPO-positive	14	22	40
Protease 3-positive	2	4	5
Complement 3 (g/l)	0.68 (0.44 to 1.18)	0.95 (0.06 to 1.83)	0.80 (0.36 to 1.99)
Serum creatinine (μmol/l)	736.5 (147.6 to 1,316) ^##^,*	165.0 (35.2 to 812.4)	381.1 (39.3 to 881)
24-hour proteinuria (g/24 hours)	0.47 (0.17 to 3.58)	0.83 (0.3 to 6.4)	1.80 (0.01 to 7.8)

Data presented as number or median (range). AAV, anti-neutrophil cytoplasmic antibody-associated vasculitis; MPO, myeloperoxidase. ^##^Difference between the crescentic-positive AAV patients and crescentic-negative AAV patients (*P* < 0.01). *Difference between the crescentic-positive AAV patients and AAV patients without a renal biopsy (*P* < 0.05).Note: Each group had one patient shown double positive MPO- and PR3- ANCA in crescentic-positive AAV and crescentic-negative AAV, respectively.

### Reagents

Fluorescein isothiocyanate (FITC) anti-human CD16 was purchased from Biolegend (San Diego, CA, USA). Anti-human LAMP-2 mouse monoclonal antibody (H4B4), anti-human LAMP-2 mouse monoclonal antibody-FITC, anti-human PR3 mouse monoclonal antibody-FITC (WGM2), anti-human MPO mouse monoclonal antibody-FITC (266.6 K2), anti-LL37 (pAbC14), anti-IFNα (pAbFL-189) and normal mouse IgG1 were purchased from Santa Cruz (Heidelberg, Germany). Anti-human histone H3 rabbit polyclonal antibody was purchased from Abcam (Cambridge, UK). All secondary antibodies conjugated with fluorescence were purchased from ZSBio (Beijing, China). Phorbol 12-myristate 13-acetate was purchased from Sigma-Aldrich (St Louis, MO, USA). HBSS (without Ca^2+^ and Mg^2+^), RPMI-1640 medium (phenol red-free) and penicillin/streptomycin solution were purchased from Invitrogen (San Diego, CA, USA). The 12-mm round glass cover slips were purchased from Thermo Fisher Scientific (Waltham, MA, USA). Polymorphprep™ was purchased from Axis-Shield (Oslo, Norway). The red blood cell lysis buffer was purchased from Roche Diagnostics (Mannheim, Germany). The immunostaining fix solution, immunostaining blocking buffer, immunostaining primary antibody dilution buffer, immunofluorescence staining secondary antibody dilution buffer and anti-fade mounting medium were purchased from Beyotime (Shanghai, China). Anti-human BDCA2 mouse monoclonal antibody-FITC (130-090-510) and anti-human BDCA2 mouse monoclonal antibody-PE (130-090-511) were purchased from Miltenyi Biotec (Bergisch Gladbach, Germany). The ELISA kits for LL37 (HK321) and IFNα (3423-1A-20) were purchased from Hycult Biotech (Uden, Netherlands) and MabTech (Nacka Strand, Sweden), respectively.

### Isolation of neutrophils

Human neutrophils were isolated from HCs or patients with PiCGN by density centrifugation using Polymorphprep™ and red blood cell lysis buffer [13]. Briefly, 5 ml blood containing ethylenediamine tetraacetic acid was layered onto 5 ml Polymorphprep™. After 35 minutes of centrifugation at 500 g, the neutrophils were separated from the polymorphonuclear leukocyte-rich pellet. Residual erythrocytes were eliminated by red blood cell lysis. The neutrophil purity was routinely ~95%, as assessed by forward-scatter and side-scatter flow cytometric analyses [8]. Unless otherwise stated, the cells were resuspended in RPMI medium (phenol red-free) supplemented with 1% penicillin/streptomycin. Then, 5×10^6^ neutrophils/ml were seeded onto tissue culture plates for culture and 5×10^5^ neutrophils/ml were seeded onto glass coverslips for immunofluorescence staining. The cells were incubated at 37°C in the presence of 5% CO_2_.

### Enzyme-linked immunosorbent assay

The levels of LL37 and IFNα from sera or cell culture supernatants were quantified by ELISA according to the protocols provided by the manufacturers. The sensitivity was 0.1 ng/ml for LL37 and 7.8 pg/ml for IFNα.

### Scanning electron microscopy

Freshly purified neutrophils were allowed to adhere to glass coverslips in RPMI-1640 medium. After incubation for 30 minutes at 37°C in 5% CO_2_, the cells were stimulated with 20 μg/ml LAMP-2 antibody or left untreated. The neutrophils were then fixed in 2.5% glutaraldehyde for 2 hours at 4°C. After washing with physiological saline three times, the samples were dehydrated through a graded ethanol series. The cover slips were then transferred into a critical point dryer and dried. The surface of the specimen was coated with a 5 nm platin/carbon layer using a thin layer evaporator. The samples were then viewed with a scanning electron microscope.

### Immunofluorescence staining and detection by confocal microscopy

Immunofluorescence staining was performed on frozen sections of kidney needle biopsies from patients with PiCGN as described previously [8]. Briefly, after fixation in paraformaldehyde, the specimens were incubated with anti-LL37 (pAbC14), anti-histone H3 (pAbab8284), anti-IFNα (pAbFL-189) and anti-BDCA-2 (mAbCD303) antibodies or isotype control, followed by the appropriate secondary antibodies. DNA was stained with 4′,6-diamidino-2-phenylindole. The neutrophils were seeded onto lysinated glass slides in a 24-well cell culture plate and incubated for 1 hour in a CO_2_ incubator at 37°C. The cells were left unstimulated or were stimulated with either 100 nM phorbol 12-myristate 13-acetate or 20 μg/ml LAMP-2 antibody for up to 180 minutes at 37°C in 5% CO_2_. Subsequently, the cells were fixed and permeabilised. After rehydration with phosphate-buffered saline at room temperature, the cells were incubated with blocking buffer overnight at 4°C, and then the specimens were incubated with fluorescently labelled antibodies. The chromatin was stained with an anti-histone H3 rabbit antibody. The images were acquired as projections of a confocal stack.

### Statistical analyses

The experiments were performed in triplicate at least three separate times. Data are presented as the mean ± standard error of the mean and were analysed using GraphPad Prism software 5 (GraphPad Software, San Diego, CA, USA). Where appropriate, either two-tailed Student’s *t* tests or the Kruskal–Wallis and Mann–Whitney *U* tests were used. Differences were considered significant at *P* <0.05.

## Results

### Serum LL37 levels are increased in AAV patients

**Figure 1:**
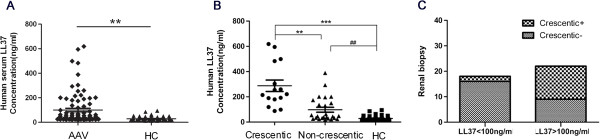
**High serum LL37 levels in anti-neutrophil cytoplasmic antibody-associated vasculitis patients. (A)** Anti-neutrophil cytoplasmic antibody-associated vasculitis (AAV) sera contained higher levels of LL37 than the healthy control (HC) sera (*U* = 829.00, **P <0.01). **(B)** AAV patients with crescentic glomerulonephritis (GN) showed higher levels of LL37 as compared with the noncrescentic GN AAV patients (**P <0.01) and the HCs (***P <0.001). Serum levels of LL37 in the patients without crescentic GN were also significantly increased compared with those in the HCs (^##^*P* <0.01). **(C)** Patients with high LL37 expression (>100.3 ng/ml) had a greater risk of having crescentic GN than patients with low LL37 expression (<100.3 ng/ml) (χ^2^ = 9.724, *P* = 0.002).

To analyse the correlation between the serum levels of LL37 and AAV, we first performed ELISAs to determine the LL37 levels in 136 serum samples collected from 85 unrelated patients with documented AAV (50 females, 35 males; female/male ratio 10:7) (Table 1) and 51 HCs. The AAV sera contained higher levels of LL37, with a mean concentration of 100.3 ng/ml, as compared with the HC sera, which showed a mean concentration of 28.53 ng/ml (*U* = 829.00, *P* <0.01; Figure 1A). Serum levels of LL37 >100.3 ng/ml were arbitrarily considered to represent high levels, and those <100.3 ng/ml were considered to represent low levels. Among the 40 patients with renal biopsy, as shown in Figure 1B, the renal tissue of patients with crescentic GN showed higher levels of LL37 as compared with those from patients with noncrescentic GN (*P* <0.01) and HCs (*P* <0.001). The serum levels of LL37 in patients without crescentic GN were also significantly higher than those in HCs (*P* <0.01). Forty patients underwent renal biopsy and 22 of them expressed high serum levels of LL37 (>100.3 ng/ml), and 13 of these high-expression patients had crescentic GN. In contrast, only two of the 18 patients expressing low serum levels of LL37 (<100.3 ng/ml) had crescentic GN (χ^2^ = 9.724, *P* = 0.002; Figure 1C). These results indicated that patients with high LL37 expression had a greater risk of having crescentic GN as compared with patients with low LL37 expression.

### Serum IFNα levels are increased in AAV patients

**Figure 2:**
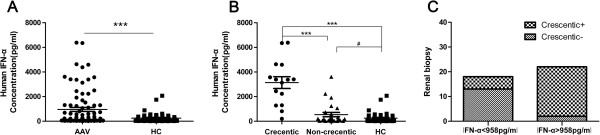
**High serum IFNα levels in anti-neutrophil cytoplasmic antibody-associated vasculitis patients. (A)** Anti-neutrophil cytoplasmic antibody-associated vasculitis (AAV) sera contained higher levels of interferon alpha (IFNα) than the healthy control (HC) sera (*U* = 1,438.00, ***P = 0.000). **(B)** AAV patients with crescentic glomerulonephritis (GN) showed higher levels of IFNα as compared with the noncrescentic GN AAV patients (***P <0.001) and the HCs (***P <0.001). Serum levels of IFNα in the patients without crescentic GN were also significantly higher than those in the HCs (^#^*P* < 0.05). **(C)** Patients with high IFNα expression (>958.5 pg/ml) had a greater risk of having crescentic GN than patients with low IFNα expression (<958.5 pg/ml) (χ^2^ = 16.835, *P* = 0.000).

We also performed ELISAs to analyse the serum IFNα levels in 136 serum samples collected from the aforementioned 85 patients. We found that the AAV sera contained higher levels of IFNα, with a mean concentration of 958.5 pg/ml, compared with the HC sera, which showed a mean concentration of 252.1 pg/ml (*U* = 1438.00, *P* = 0.000; Figure 2A).

Serum levels of IFNα >958.5 pg/ml were arbitrarily considered to represent high levels, whereas those <958.5 pg/ml were considered to represent low levels. Among the 40 patients with renal biopsy, as shown in Figure 2B, 15 crescentic GN patients showed high-level IFNα expression as compared with 25 of the noncrescentic GN patients (*P* <0.001) and the HCs (*P* <0.001). The IFNα serum level of the patients without crescentic GN were also significantly higher than those in HCs (*P* <0.05). Of the 40 patients who provided a renal biopsy, 22 showed high levels of IFNα (>958.5 pg/ml), and 13 of these 22 patients had crescentic GN. In contrast, only two of the 18 patients expressing low serum levels of IFNα (<958.5 pg/ml) had crescentic GN (χ^2^ = 16.835, *P* = 0.000; Figure 2C). These results indicated that patients expressing high IFNα levels had a greater risk of crescentic formation in the glomerulus in comparison with patients expressing low levels of IFNα.

### Correlation of the serum LL37 and IFNα levels with the serological parameters

**Figure 3:**
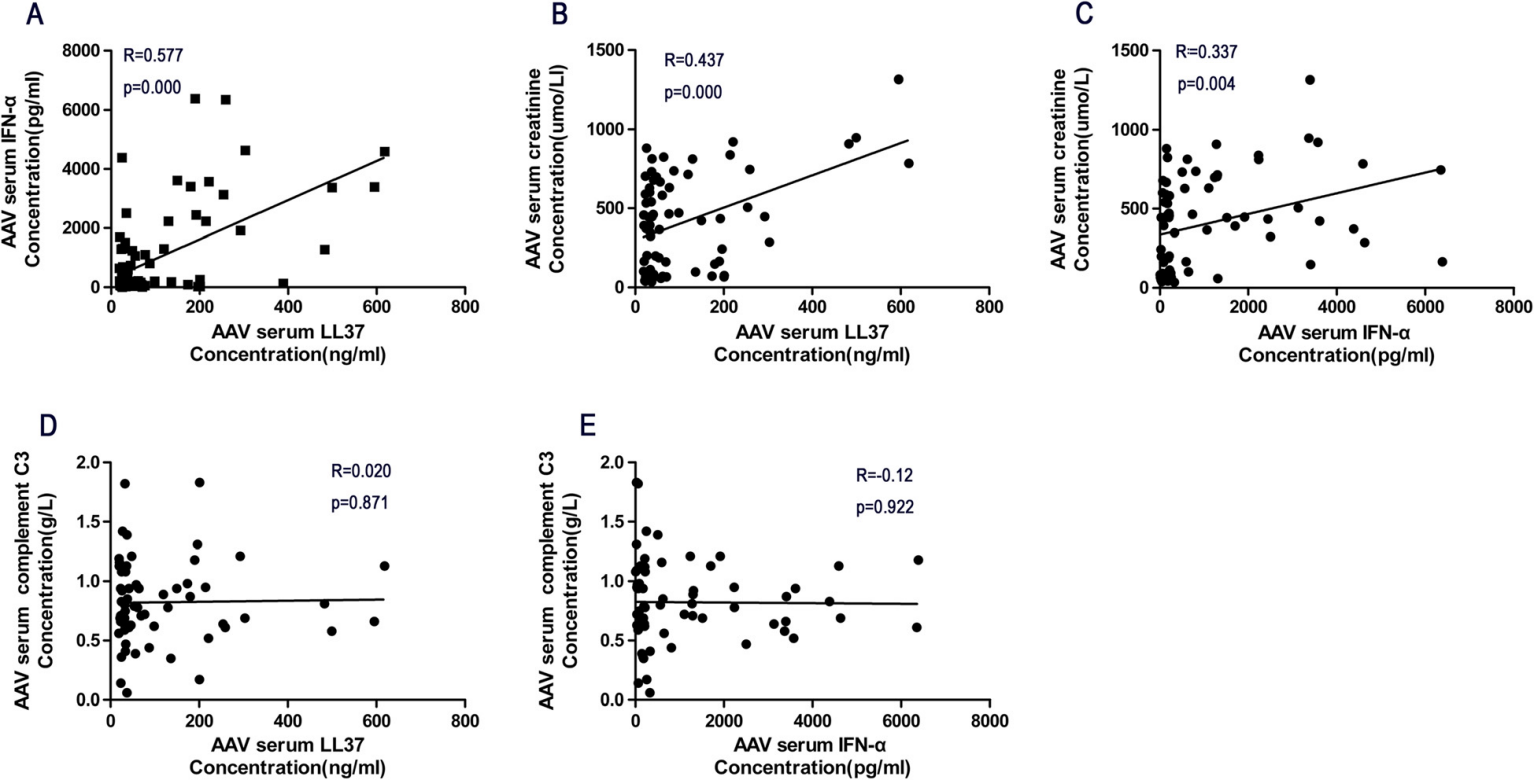
**Correlation of serum LL37 and IFNα levels with serological parameters. (A)** The serum LL37 levels were positively correlated with the interferon alpha (IFNα) levels (*R* = 0.577, *P* = 0.000). **(B)**, **(C)** Both the serum LL37 (*R* = 0.437, *P* = 0.000) and IFNα (*R* = 0.337, *P* = 0.004) levels showed a positive correlation with the serum creatinine levels, **(D)**, **(E)** but not with the complement C3 levels (LL37: *R* = 0.020, *P* = 0.871; IFNα: *R* = -0.12, *P* = 0.922). AAV, anti-neutrophil cytoplasmic antibody-associated vasculitis.

Given that the LL37 and IFNα serum levels were increased in AAV patients, particularly in those with crescentic GN, we further investigated whether the serum levels of LL37 were correlated with the IFNα levels and whether the levels of LL37 and IFNα were correlated with the serum levels of creatinine and complement C3. We found a positive correlation between the serum LL37 and IFNα levels (*R* = 0.577, *P* = 0.000; Figure 3A) as well as significant positive correlations between the serum levels of LL37 and creatinine (*R* = 0.437, *P* = 0.000; Figure 3B) and between the serum levels of IFNα and creatinine (*R* = 0.337, *P* = 0.004; Figure 3C). However, neither LL37 nor IFNα showed any correlation with complement C3 (LL37: *R* = 0.020, *P* = 0.871; IFNα: *R* = -0.12, *P* = 0.922) (Figure 3D,E). To further investigate whether LL37 and IFNα reflect systemic inflammation, we analysed the sera levels of LL-37 and IFNα before and after treatment (*n* = 7). We found that both LL-37 and IFNα levels had degraded after immunosuppressive therapy (Additional file 1).

### Release of LL37 and IFNα in the kidney

**Figure 4:**
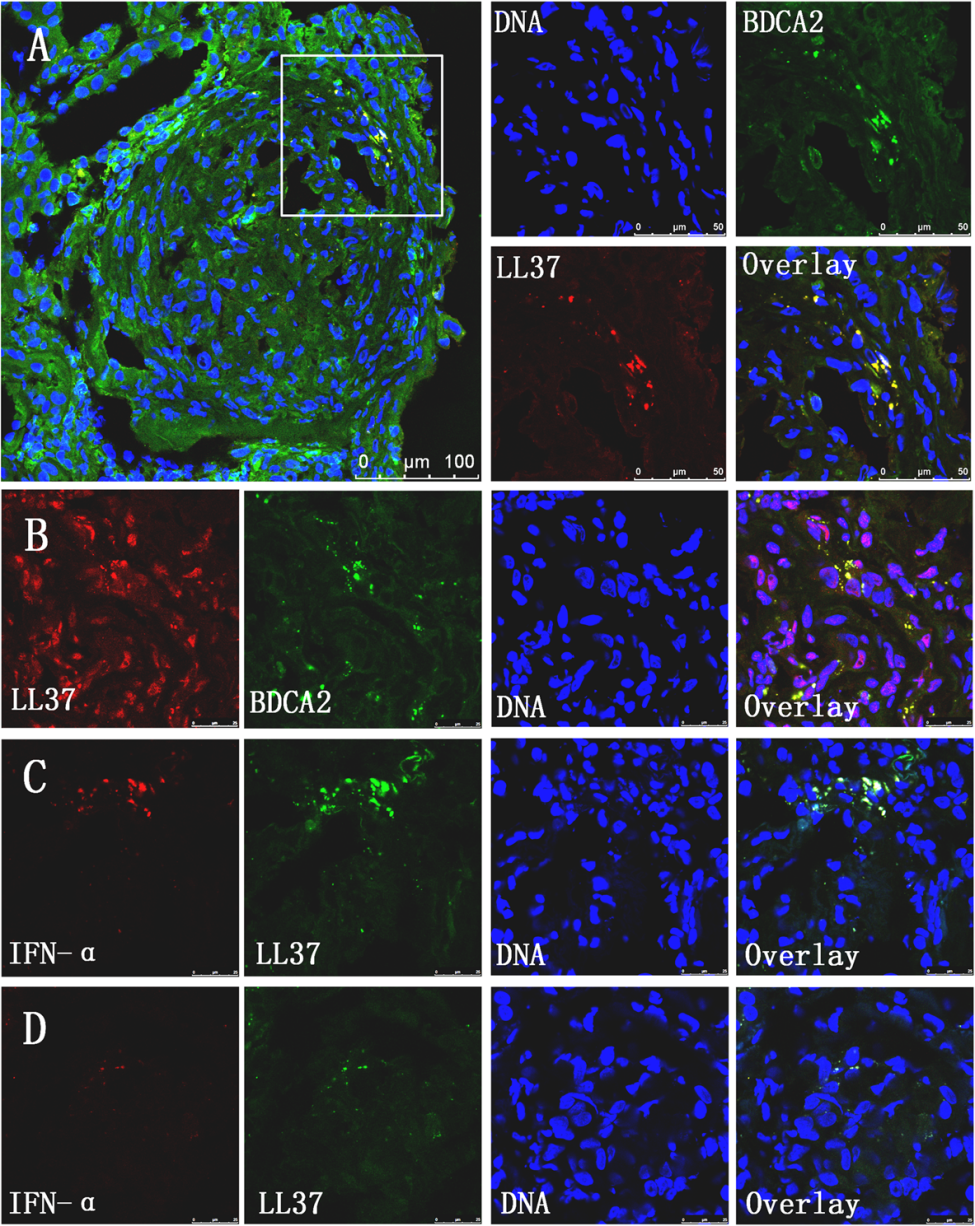
**Expression of LL37, IFNα and BDCA2 in anti-neutrophil cytoplasmic antibody-associated vasculitis patients with crescentic glomerulonephritis. (A)**, **(B)** Representative images showing the co-localisation of LL37 (red) and BDCA2, a marker of plasmacytoid dendritic cells (green), in the glomeruli of frozen renal biopsy sections after immunofluorescence staining. **(C)** Co-localisation of interferon alpha (IFNα; red) and LL37 (green) in renal biopsy sections from anti-neutrophil cytoplasmic antibody-associated vasculitis (AAV) patients with crescentic glomerulonephritis (GN). **(D)** Co-localisation of IFNα (red) and LL37 (green) in renal biopsy sections from AAV patients without crescentic GN.

To assess the roles of LL37 and IFNα in renal inflammation, we evaluated the presence and localisation of LL37 and IFNα in renal biopsies from AAV patients with crescentic GN. We found that the kidney tissues from AAV patients showed strong expression of LL37, especially in Bowman’s capsule. We also found that LL37 expression co-localised with neutrophil and pDC (stained for BDCA2) infiltrates in the affected glomeruli and the interstitium (Figure 4A,B), suggesting that LL37 expression occurs predominantly during crescentic GN. As autoinflammatory conditions such as psoriasis can be driven by pDCs, which produce large amounts of IFNα, we next examined the expression of IFNα in the kidney tissues. Immunostaining revealed the co-localisation of IFNα and LL37 in the kidney tissues from AAV patients (Figure 4C). However, there were only mild immunofluorescence signals stained with IFNα and LL37 in the renal tissues from patients without crescents (Figure 4D), suggesting that LL37 may mediate pDC activation to produce IFNα in renal local inflammatory in AAV.

### Release of auto-antigens and cathelicidin LL37 from neutrophil extracellular traps

**Figure 5:**
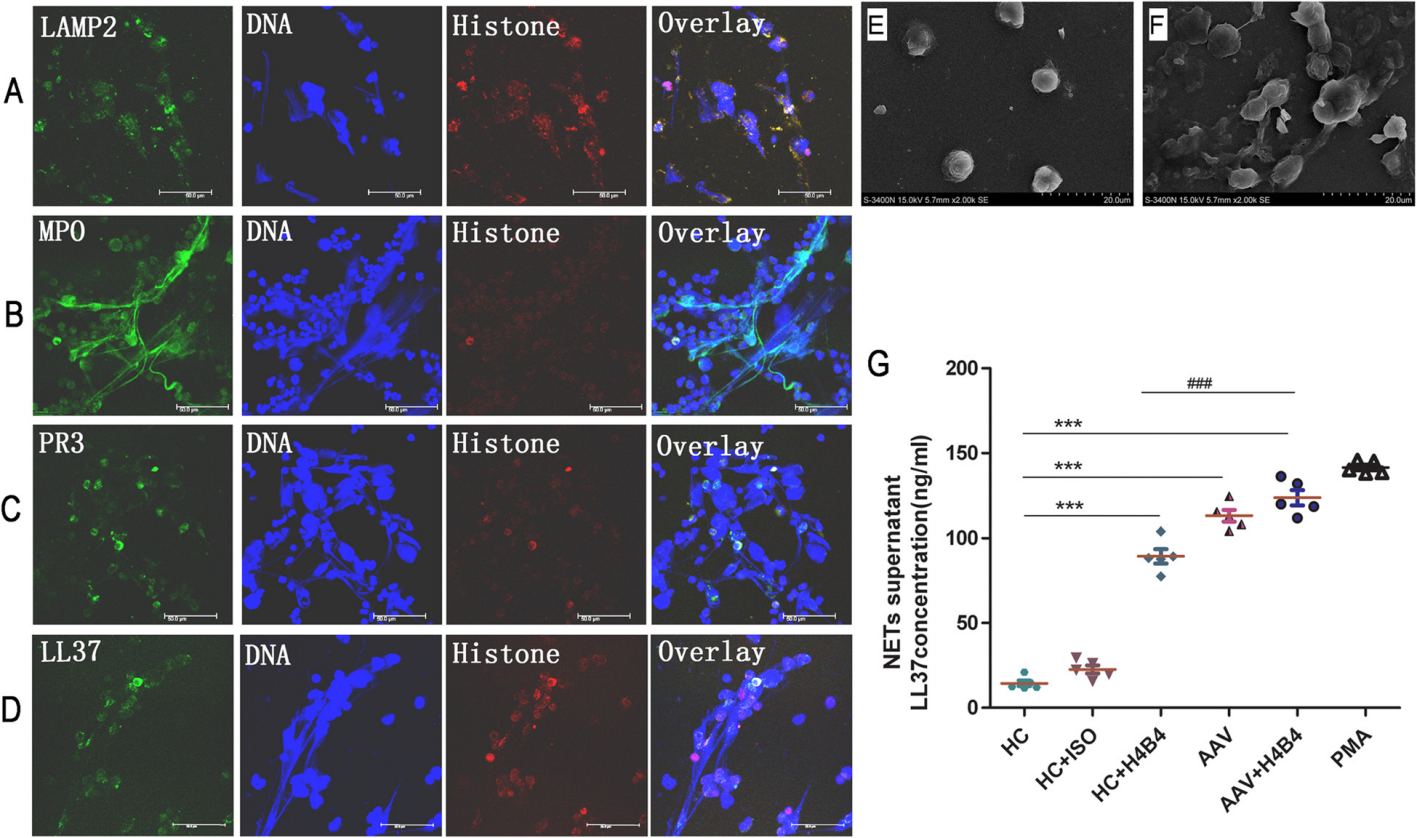
**Anti-neutrophil cytoplasmic antibody**-**induced neutrophil extracellular traps release auto-antigens and LL37.** Neutrophils were incubated with anti-hLAMP2-IgG (H4B4, 20 μg/ml) for 180 minutes with buffer from a healthy donor. Auto-antigens for **(A)** LAMP-2, **(B)** myeloperoxidase (MPO), **(C)** proteinase-3 (PR3) and **(D)** LL37 were examined (green) in the neutrophil extracellular traps (NETs). Histones were stained with an H3 rabbit polyclonal antibody (red), and DNA was stained with 4′,6-diamidino-2-phenylindole (blue). Neutrophil features were observed under scanning electron microscopy for **(E)** healthy controls (HCs) and **(F)** anti-neutrophil cytoplasmic antibody-associated vasculitis (AAV) patients after incubation for 180 minutes *in vitro*. **(G)** Quantification of the supernatant levels of LL37 by enzyme-linked immunosorbent assay: supernatant of neutrophils from healthy control incubated with anti-hLAMP2-IgG (HC+H4B4), neutrophils from AAV patients (AAV) and neutrophils from AAV patients incubated with anti-hLAMP2-IgG (AAV+H4B4)groups showed higher levels of LL37 compared with HC group (***P <0.001), respectively. Supernatant of neutrophils from AAV +H4B4 group showed higher levels of LL37 compared with the HC + H4B4 group (^###^P <0.001). ISO; PMA, phorbol 12-myristate 13-acetate.

ANCA plays a critical role in the vascular damage associated with AAV [14]. Kessenbrock and colleagues reported strong NET formation in patients with AAV [8], and the presence of anti-LAMP-2 antibodies has been suggested to represent a new subtype of ANCA, with a high prevalence in PiCGN [2,4,15]. Therefore, we asked whether targeted auto-antigens and antimicrobial peptides would be present in NETs stimulated by anti-LAMP-2-IgG. As expected, the immunofluorescent analysis of NETs revealed that LAMP-2 co-localised with extracellular chromatin fibres (Figure 5A). We also observed that PR3 and MPO were expressed within the NETs (Figure 5B,C), as reported previously [8], and we found that LL37 expression was enriched in parts of the extracellular chromatin fibres (Figure 5D). After culturing for 180 minutes, neutrophils isolated from PiCGN patients and HCs showed the typical features of NETosis, including web-like structures, as visualised by scanning electron microscopy (Figure 5E,F). To investigate the release of LL37 in the NET supernatant, neutrophils isolated from AAV patients or HCs were treated with anti-LAMP-2-IgG (AAV + H4B4, *n* = 5; HC + H4B4, *n* = 5), an isotype control (HC + ISO, *n* = 5) or phorbol 12-myristate 13-acetate as a positive control (*n* = 5) [8]. After 180 minutes, the supernatant was collected and measured by ELISA, and the results indicated that the HC + H4B4 group supernatant showed higher levels of LL37 as compared with the HC or HC + ISO groups (*P* <0.001). Moreover, the AAV + H4B4 supernatant showed higher levels of LL37 compared with the HC + H4B4 supernatant (*P* <0.001) (Figure 5G). No IFNα was detected in the supernatant by ELISA. We observed MPO, a marker of neutrophils, showing co-localisation with LL37, IFNα and BDCA-2 in AAV patients with crescentic GN (Additional file 2). To further clarify the source IFNα, pDCs were isolated by magnetically activated cell sorting using the BDCA-4 dendritic cell isolation kit (Miltenyi Biotec). pDCs (5 × 10^4^/ml) were incubated with supernatants from neutrophils treated as above mentioned, with 3 μg/ml ODN 2216 pDCs as positive control for 24 hours. Supernatants were harvested to detect the level of IFNα and LL-37 by ELISA. We found that IFNα was released by pDC incubation with supernatants of anti-LAMP-2 antibody-treated neutrophils and ODN2216 for 24 hours (Additional file 3). No LL-37 was released from pDC.

## Discussion

The present study is the first to demonstrate that LL37 levels are significantly increased in AAV patients, especially in those with PiCGN. The serum levels of LL37 and IFNα were positively correlated with crescentic formation and also positively correlated with the serum levels of creatinine. Furthermore, we found evidence that LL37 and IFNα expression were co-localised in inflammatory kidney tissue. There were also higher levels of LL37 released in AAV patients as compared with HCs, as determined by NET formation *in vitro*.

PiCGN is a serious manifestation in patients with AAV and can rapidly progress to end-stage renal failure. Despite the large number of studies, the pathogenesis of PiCGN has not been fully clarified. Recently, the constituents derived from NETs, such as high-mobility group box-1 protein, have been shown to play an important role in the renal pathology of systemic lupus erythematosus patients, potentially reflecting both local renal inflammation and systemic inflammation [16,17]. The role of cathelicidin LL37 released from these NETs has been demonstrated in autoimmune and chronic inflammatory diseases, especially those with renal manifestations such as systemic lupus erythematosus and psoriasis [6,7]. However, no studies have been performed to evaluate the serum levels of LL37 to determine whether LL37 expression is a reflection of systemic and/or renal local inflammation in AAV patients. We found that the serum levels of LL37 were significantly increased in AAV patients, especially in those with PiCGN compared with patients without crescentic formation and HCs. In line with a previous study, the serum levels of IFNα were significantly increased in patients with active AAV [8], and we also found that the serum levels of IFNα were increased similarly in AAV patients with PiCGN. However, the origin of LL37 – that is, whether it is produced outside the kidney or locally within the inflamed kidney – remains unresolved.

The precise events leading to glomerular inflammation and damage in ANCA-related PiCGN remain poorly understood. pDCs comprise a DC population that is highly specialised to sense viral and certain microbial infections. LL37 plays a key role in converting self-DNA into a stimulatory ligand for pDCs [7], and the LL37 expression in renal biopsy tissues from PiCGN patients was strong. BDCA-2 is a pDC-specific marker [18], and we found that BDCA2 co-localised with LL37 in the glomeruli and interstitium. Furthermore, a previous report indicating that pDCs produce large amounts of IFNα in the presence of DNA and LL37 [7] suggests that IFNα may be released from pDCs in renal tissue. We also found that LL37 co-localised with IFNα in PiCGN renal biopsies, and these results indicate that pDCs are activated and participate in the inflammatory response in the kidney. As kidney injury coupled with the expression of LL37 may elicit the sustained accumulation and activation of pDCs in the kidney, LL37 antagonists may potentially be developed as therapies for PiCGN and other chronic inflammatory diseases, whereas LL37 itself may potentially serve as a vaccine adjuvant [7].

Neutrophils are considered the mainstay of the cellular innate immune response. Many data have shown that neutrophils are not only basic players and mediators of innate immunity but are also involved in the activation, regulation and effector functions of adaptive immune cells, such as dendritic cells, B cells and T cells [19,20]. In addition, there has been increased attention on extracellular neutrophil traps in recent years with regard to the pathogenesis of diverse inflammatory and autoimmune diseases [21-26]. Summers and colleagues reported that neutrophil recruitment may play an important role in experimental anti-MPO crescentic GN [27], and recent research has reported that autoantibodies against human LAMP-2 represent a new ANCA subtype that can be induced by infection with fimbriated bacteria, which occurs at a high prevalence in PiCGN cases [2,3]. We found that anti-LAMP-2-IgG triggered NET formation and that targeted auto-antigens for LAMP-2, PR3, MPO and LL37 were present within those NETs. There were also higher levels of LL37 released in AAV patients compared with HCs, as determined by NET formation *in vitro*. This result demonstrates that ANCA can activate neutrophil-released auto-antigens through NET formation. This process results in the expression of LAMP-2, MPO, PR3 and LL37 or high-mobility group box-1 protein, which all have the characteristic dual capacity to mobilise and activate antigen-presenting cells, thereby further inducing the activation of immune cells [28]. These observations indicate that ANCA may perpetuate a vicious circle of NET production that maintains the delivery of endogenous danger signals to the immune system.

The present study provides evidence that LL37 expression is increased not only in the sera but also at the site of local renal inflammation in AAV. The serum levels of LL37 could thus reflect both local and systemic inflammation. However, further studies are needed to evaluate the clinical significance of LL37 in a larger sample as well as its value as a biomarker in AAV patients with renal involvement.

## Conclusions

Together, our findings indicated that the serum levels of LL37 and IFNα levels were increased in AAV patients, particularly those with crescentic GN. These increases in the serum LL37 and IFNα levels were correlated with crescentic formation. Accordingly, the crescentic patients showed evidence of LL37 and IFNα expression in their local inflammatory renal tissue. Taken together, these data suggest that LL37 may play an important role in the renal pathology of AAV patients.

## Abbreviations

AAV: Anti-neutrophil cytoplasm antibody-associated vasculitis; ANCA: Anti-neutrophil cytoplasm antibody; C3: Complement 3; ELISA: Enzyme-linked immunosorbent assay; FITC: Fluorescein isothiocyanate; GN: Glomerulonephritis; HC: Healthy control; IFNα: Interferon alpha; LAMP-2: Lysosomal membrane protein-2; MPO: Myeloperoxidase; NET: Neutrophil extracellular trap; pDC: Plasmacytoid dendritic cell; PiCGN: Pauci-immune crescentic glomerulonephritis; PR3: Proteinase-3.

## Competing interests

The authors declare that they have no competing interests.

## Authors’ contributions

JBZ, ASG, YZW and FHY were involved in all aspects of the study conception, design and direction. JBZ, ASG, YZW, YZ, FHY, WWS, ST, LS, LW, and SWY were involved in the data acquisition, the analysis and interpretation of the results and drafted the manuscript. YZ, WWS, ST, LS, LW and XJG performed the cell isolation, YZ, WWS, XJG, JYL and LYZ carried out the measurements of NET, LL37 and IFNα. YZ, ST, WWS, JHZ and YJH carried out immunofluorescence staining and assays. All authors read and approved the final manuscript.

## Supplementary Material

Additional file 1: Figure S1 Showing the levels of serum LL-37 and IFNα before and after treatment. Serum levels of LL37 (A) and IFNα (B) were significantly decreased after immunosuppressive therapy than before treatment (*n* = 7, *P < 0.05). Click here for file

Additional file 2: Figure S2 Showing expression of MPO, LL37, IFNα and BDCA2 in AAV patients with crescentic GN. (A) Representative images showing the co-localisation of MPO (green) and LL37 (red) in the glomeruli of frozen renal biopsy sections after immunofluorescence staining. (B) Co-localisation of MPO (green) and IFNα (red) in renal biopsy sections from AAV patients with crescentic GN. (C) Co-localisation of MPO (green) and BDCA2 (red) in renal biopsy sections from AAV patients without crescentic GN. Click here for file

Additional file 3: Figure S3 Showing IFNα released from pDC. None IFNα were detected in the supernatants from neutrophils treated with or without anti-LAMP-2 antibodies (H4B4) for 3 hours. The mean level in the supernatants of IFNα was 7,357 pg/ml from pDCs incubated with ODN2216 groups, and was 866 pg/ml from pDCs incubated with supernatants of anti-LAMP-2 antibody-treated neutrophils. The level was under-detectible in supernatants from pDCs treated with medium. Click here for file

## References

[B1] Jennette JC Falk RJ Small-vessel vasculitis N Engl J Med 1997 15 1512 1523 10.1056/NEJM199711203372106 9366584

[B2] Kain R Tadema H McKinney EF Benharkou A Brandes R Peschel A Hubert V Feenstra T Sengolge G Stegeman C Heeringa P Lyons PA Smith KG Kallenberg C Rees AJ High prevalence of autoantibodies to hLAMP-2 in anti-neutrophil cytoplasmic antibody-associated vasculitis J Am Soc Nephrol 2012 15 556 566 10.1681/ASN.2011090920 22323643 PMC3294304

[B3] Kain R Exner M Brandes R Ziebermayr R Cunningham D Alderson CA Davidovits A Raab I Jahn R Ashour O Spitzauer S Sunder-Plassmann G Fukuda M Klemm P Rees AJ Kerjaschki D Molecular mimicry in pauci-immune focal necrotizing glomerulonephritis Nat Med 2008 15 1088 1096 10.1038/nm.1874 18836458 PMC2751601

[B4] Salama AD Pusey CD Shining a LAMP on pauci-immune focal segmental glomerulonephritis Kidney Int 2009 15 15 17 10.1038/ki.2009.123 19387471

[B5] Frasca L Lande R Role of defensins and cathelicidin LL37 in auto-immune and auto-inflammatory diseases Curr Pharm Biotechnol 2012 15 1882 1897 10.2174/138920112802273155 22250708

[B6] Garcia-Romo GS Caielli S Vega B Connolly J Allantaz F Xu Z Punaro M Baisch J Guiducci C Coffman RL Barrat FJ Banchereau J Pascual V Netting neutrophils are major inducers of type I IFN production in pediatric systemic lupus erythematosus Sci Transl Med 2011 15 73ra20 10.1126/scitranslmed.3001201 21389264 PMC3143837

[B7] Lande R Gregorio J Facchinetti V Chatterjee B Wang YH Homey B Cao W Su B Nestle FO Zal T Mellman I Schroder JM Liu YJ Gilliet M Plasmacytoid dendritic cells sense self-DNA coupled with antimicrobial peptide Nature 2007 15 564 569 10.1038/nature06116 17873860

[B8] Kessenbrock K Krumbholz M Schonermarck U Back W Gross WL Werb Z Grone HJ Brinkmann V Jenne DE Netting neutrophils in autoimmune small-vessel vasculitis Nat Med 2009 15 623 625 10.1038/nm.1959 19448636 PMC2760083

[B9] Nizet V Ohtake T Lauth X Trowbridge J Rudisill J Dorschner RA Pestonjamasp V Piraino J Huttner K Gallo RL Innate antimicrobial peptide protects the skin from invasive bacterial infection Nature 2001 15 454 457 10.1038/35106587 11719807

[B10] Knight JS Kaplan MJ Lupus neutrophils: ‘NET’ gain in understanding lupus pathogenesis Curr Opin Rheumatol 2012 15 441 450 10.1097/BOR.0b013e3283546703 22617827

[B11] Chamilos G Gregorio J Meller S Lande R Kontoyiannis DP Modlin RL Gilliet M Cytosolic sensing of extracellular self-DNA transported into monocytes by the antimicrobial peptide LL37 Blood 2012 15 3699 3707 10.1182/blood-2012-01-401364 22927244 PMC3488884

[B12] Jennette JC Falk RJ Andrassy K Bacon PA Churg J Gross WL Hagen EC Hoffman GS Hunder GG Kallenberg CG Nomenclature of systemic vasculitides, Proposal of an international consensus conference Arthritis Rheum 1994 15 187 192 10.1002/art.1780370206 8129773

[B13] Brinkmann V Laube B Abu Abed U Goosmann C Zychlinsky A Neutrophil extracellular traps: how to generate and visualize them J Vis Exp 2010 15 1724 20182410 10.3791/1724 PMC3125121

[B14] Bosch X LAMPs and NETs in the pathogenesis of ANCA vasculitis J Am Soc Nephrol 2009 15 1654 1656 10.1681/ASN.2009060616 19608698

[B15] Bosch X Mirapeix E Vasculitis syndromes: LAMP-2 illuminates pathogenesis of ANCA glomerulonephritis Nat Rev Nephrol 2009 15 247 249 10.1038/nrneph.2009.51 19384321

[B16] Abdulahad DA Westra J Bijzet J Dolff S van Dijk MC Limburg PC Kallenberg CG Bijl M Urine levels of HMGB1 in systemic lupus erythematosus patients with and without renal manifestations Arthritis Res Ther 2012 15 R184 10.1186/ar4015 22892043 PMC3580580

[B17] Abdulahad DA Westra J Bijzet J Limburg PC Kallenberg CG Bijl M High mobility group box 1 (HMGB1) and anti-HMGB1 antibodies and their relation to disease characteristics in systemic lupus erythematosus Arthritis Res Ther 2011 15 R71 10.1186/ar3332 21548924 PMC3218880

[B18] Riboldi E Daniele R Cassatella MA Sozzani S Bosisio D Engagement of BDCA-2 blocks TRAIL-mediated cytotoxic activity of plasmacytoid dendritic cells Immunobiology 2009 15 868 876 10.1016/j.imbio.2009.06.016 19577819

[B19] Mantovani A Cassatella MA Costantini C Jaillon S Neutrophils in the activation and regulation of innate and adaptive immunity Nat Rev Immunol 2011 15 519 531 10.1038/nri3024 21785456

[B20] Tillack K Breiden P Martin R Sospedra M T lymphocyte priming by neutrophil extracellular traps links innate and adaptive immune responses J Immunol 2012 15 3150 3159 10.4049/jimmunol.1103414 22351936

[B21] Brill A Fuchs TA Savchenko AS Thomas GM Martinod K De Meyer SF Bhandari AA Wagner DD Neutrophil extracellular traps promote deep vein thrombosis in mice J Thromb Haemost 2012 15 136 144 10.1111/j.1538-7836.2011.04544.x 22044575 PMC3319651

[B22] Kambas K Mitroulis I Apostolidou E Girod A Chrysanthopoulou A Pneumatikos I Skendros P Kourtzelis I Koffa M Kotsianidis I Ritis K Autophagy mediates the delivery of thrombogenic tissue factor to neutrophil extracellular traps in human sepsis PLoS One 2012 15 e45427 10.1371/journal.pone.0045427 23029002 PMC3446899

[B23] Leffler J Martin M Gullstrand B Tyden H Lood C Truedsson L Bengtsson AA Blom AM Neutrophil extracellular traps that are not degraded in systemic lupus erythematosus activate complement exacerbating the disease J Immunol 2012 15 3522 3531 10.4049/jimmunol.1102404 22345666

[B24] Megens RT Vijayan S Lievens D Doring Y van Zandvoort MA Grommes J Weber C Soehnlein O Presence of luminal neutrophil extracellular traps in atherosclerosis Thromb Haemost 2012 15 597 598 10.1160/TH11-09-0650 22318427

[B25] Sangaletti S Tripodo C Chiodoni C Guarnotta C Cappetti B Casalini P Piconese S Parenza M Guiducci C Vitali C Colombo MP Neutrophil extracellular traps mediate transfer of cytoplasmic neutrophil antigens to myeloid dendritic cells toward ANCA induction and associated autoimmunity Blood 2012 15 3007 3018 10.1182/blood-2012-03-416156 22932797

[B26] Thornton RB Wiertsema SP Kirkham LA Rigby PJ Vijayasekaran S Coates HL Richmond PC Neutrophil extracellular traps and bacterial biofilms in middle ear effusion of children with recurrent acute otitis media – a potential treatment target PLoS One 2013 15 e53837 10.1371/journal.pone.0053837 23393551 PMC3564866

[B27] Summers SA van der Veen BS O’Sullivan KM Gan PY Ooi JD Heeringa P Satchell SC Mathieson PW Saleem MA Visvanathan K Holdsworth SR Kitching AR Intrinsic renal cell and leukocyte-derived TLR4 aggravate experimental anti-MPO glomerulonephritis Kidney Int 2010 15 1263 1274 10.1038/ki.2010.327 20844472

[B28] Yang D de la Rosa G Tewary P Oppenheim JJ Alarmins link neutrophils and dendritic cells Trends Immunol 2009 15 531 537 10.1016/j.it.2009.07.004 19699678 PMC2767430

