# Supplementary figures and images for: The influence of cathelicidin LL37 in human anti-neutrophils cytoplasmic antibody (ANCA)-associated vasculitis
Source: Arthritis Res Ther. 2013 Oct 24;15(5):R161. doi: 10.1186/ar4344 (PMC3979017; doi:10.1186/ar4344)

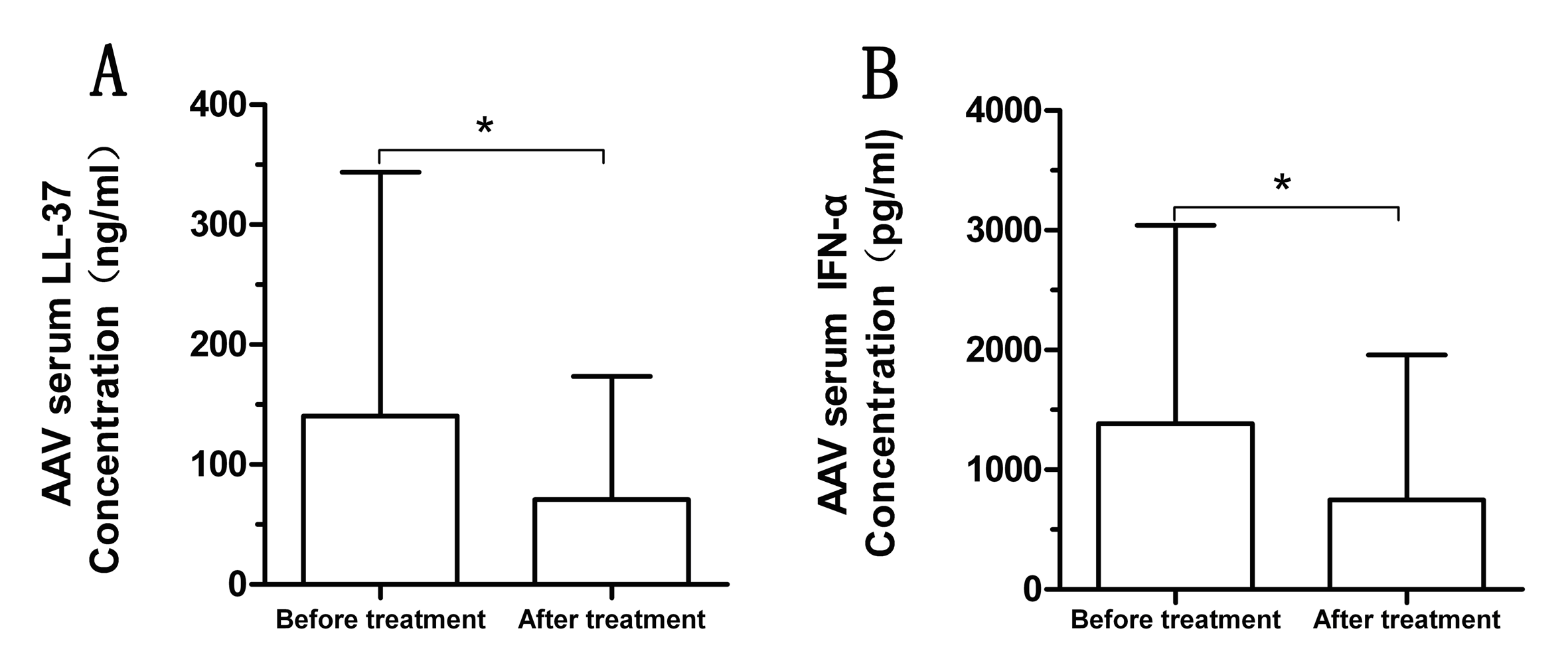

Supplement: Additional file 1: Figure S1 — Showing the levels of serum LL-37 and IFNα before and after treatment. Serum levels of LL37 (A) and IFNα (B) were significantly decreased after immunosuppressive therapy than before treatment (n = 7, *P < 0.05). [file ar4344-S1.tiff]

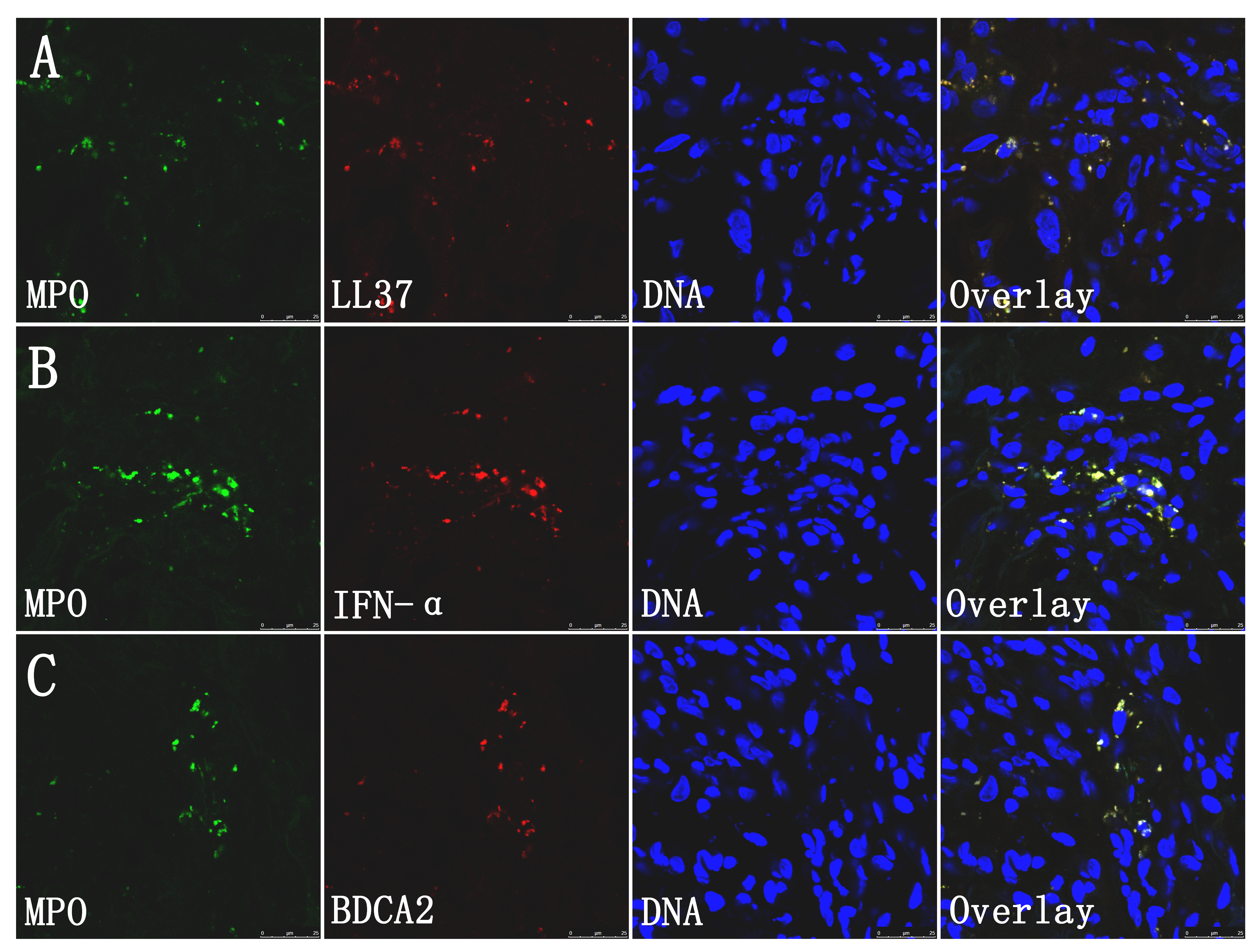

Supplement: Additional file 2: Figure S2 — Showing expression of MPO, LL37, IFNα and BDCA2 in AAV patients with crescentic GN. (A) Representative images showing the co-localisation of MPO (green) and LL37 (red) in the glomeruli of frozen renal biopsy sections after immunofluorescence staining. (B) Co-localisation of MPO (green) and IFNα (red) in renal biopsy sections from AAV patients with crescentic GN. (C) Co-localisation of MPO (green) and BDCA2 (red) in renal biopsy sections from AAV patients without crescentic GN. [file ar4344-S2.tiff]

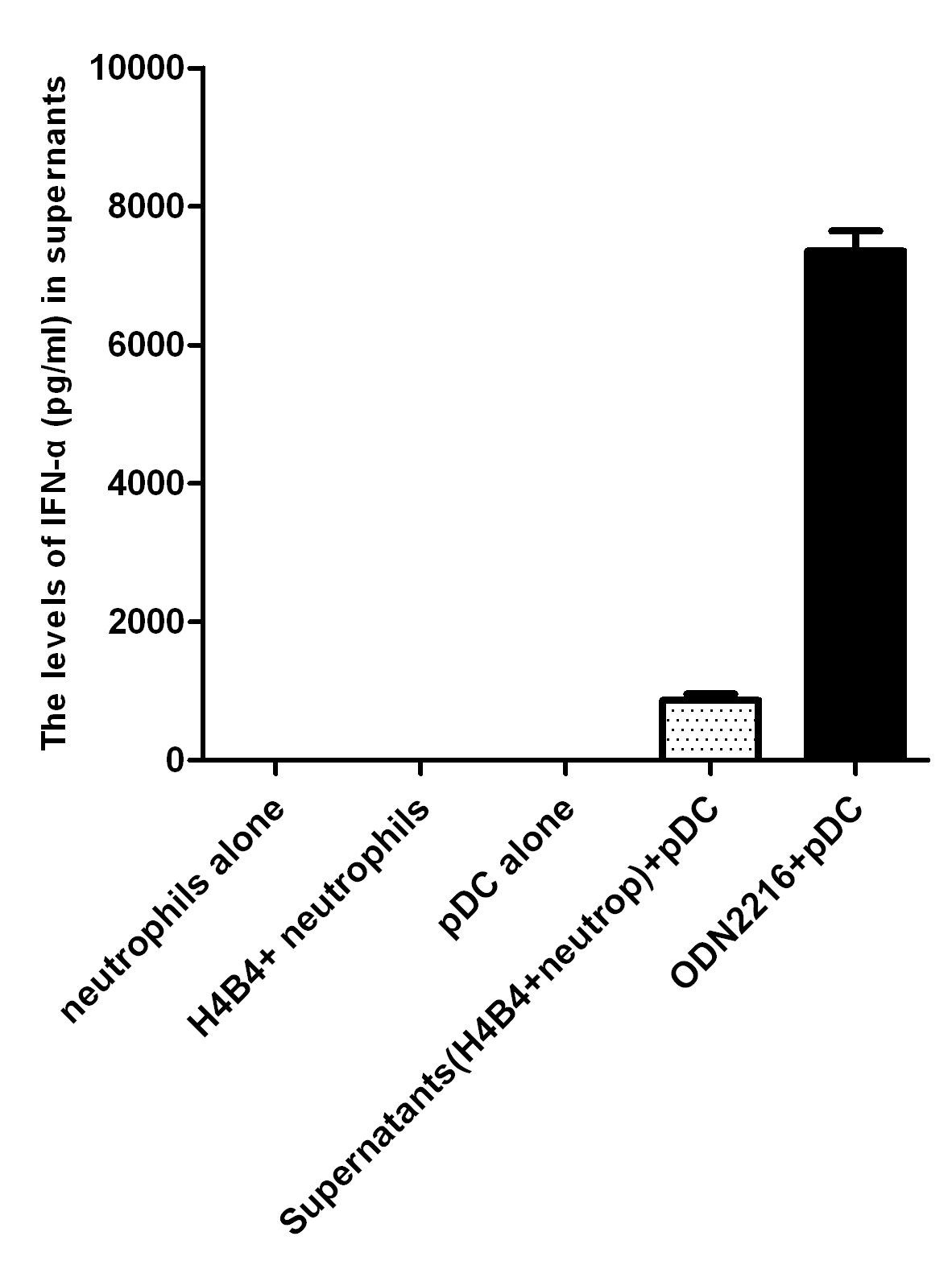

Supplement: Additional file 3: Figure S3 — Showing IFNα released from pDC. None IFNα were detected in the supernatants from neutrophils treated with or without anti-LAMP-2 antibodies (H4B4) for 3 hours. The mean level in the supernatants of IFNα was 7,357 pg/ml from pDCs incubated with ODN2216 groups, and was 866 pg/ml from pDCs incubated with supernatants of anti-LAMP-2 antibody-treated neutrophils. The level was under-detectible in supernatants from pDCs treated with medium. [file ar4344-S3.tiff]
